# Assessment and Characterisation of Ireland's Green Tides (*Ulva* Species)

**DOI:** 10.1371/journal.pone.0169049

**Published:** 2017-01-03

**Authors:** Alex H. L. Wan, Robert J. Wilkes, Svenja Heesch, Ricardo Bermejo, Mark P. Johnson, Liam Morrison

**Affiliations:** 1 Irish Seaweed Research Group, Ryan Institute and School of Natural Sciences, National University of Ireland, Galway, Co. Galway, Ireland; 2 Environmental Protection Agency, Castlebar, Co. Mayo, Ireland; 3 Earth and Ocean Sciences, Ryan Institute and School of Natural Sciences, National University of Ireland, Galway, Ireland; University of Connecticut, UNITED STATES

## Abstract

Enrichment of nutrients and metals in seawater associated with anthropogenic activities can threaten aquatic ecosystems. Consequently, nutrient and metal concentrations are parameters used to define water quality. The European Union’s Water Framework Directive (WFD) goes further than a contaminant-based approach and utilises indices to assess the Ecological Status (ES) of transitional water bodies (e.g. estuaries and lagoons). One assessment is based upon the abundance of opportunistic *Ulva* species, as an indication of eutrophication. The objective of this study was to characterise Ireland’s *Ulva* blooms through the use of WFD assessment, metal concentrations and taxonomic identity. Furthermore, the study assessed whether the ecological assessment is related to the metal composition in the *Ulva*. WFD algal bloom assessment revealed that the largest surveyed blooms had an estimated biomass of 2164 metric tonnes (w/w). DNA sequences identified biomass from all locations as *Ulva rigida*, with the exception of New Quay, which was *Ulva rotundata*. Some blooms contained significant amounts of As, Cu, Cr, Pb and Sn. The results showed that all metal concentrations had a negative relationship (except Se) with the Ecological Quality Ratio (EQR). However, only in the case of Mn were these differences significant (*p* = 0.038). Overall, the metal composition and concentrations found in *Ulva* were site dependent, and not clearly related to the ES. Nevertheless, sites with a moderate or poor ES had a higher variability in the metals levels than in estuaries with a high ES.

## Introduction

Anthropogenic activities occurring in the coastal areas can produce an array of stressors on the local biological communities. These pressures can change the aquatic conditions producing different forms of pollution (e.g. dystrophy caused by an excess of eutrophication, acidification, metal toxicity, biological invasions, and pollution by organic compounds and organic matter) that degrade the environment. This environmental degradation is particularly significant in the coastal zone, where human activities have been historically concentrated [1]. The Atlantic coast of Europe and the Mediterranean basin have been inhabited for millennia, and consequently alteration of environmental conditions and anthropogenic pressures are typically more pronounced than in other coastal areas of the world [2]. Eutrophication has been identified as a principal pressure in European marine ecosystems, and decreasing nutrient loading to these systems has been identified as the main restoration solution [3]. The effects of this pressure can also alter irradiance levels and substrate composition and consequently reduce the composition diversity of the aquatic and benthic communities [4, 5, 6]. Past studies have shown that the anthropogenic release of potentially toxic metals into the water column can be three times the amount from natural inputs [7, 8, 9]. It has been well documented that exposure to priority pollutants (e.g. arsenic, cadmium, chromium, and lead) can be a risk to both ecosystems [9] and human health [10].

To monitor coastal pollution, environmental surveys often primarily involve the measurement of physical and chemical attributes to give a sense of water quality. However, the exclusive use of such methods can give a localised and transient measurement, and overall offers little indication of the long term effects of pollutants on the benthic ecology [11]. The use of bioindicators as an ecological monitoring tool has advantages over physico-chemical indicators. The core principle of using bioindicators is that they give a direct measure of the effect of pollution on the organisms. Bioindicators could also give an indication of pollutant effects in the benthic communities once the pollutant has disappeared from the aquatic system or when levels of contaminants are too low for accurate determination to be carried out by analytical instruments [11]. Moreover, the need to carry out measurements on the bioindicator is typically less frequent when compared to direct water sampling due to movement of water bodies (e.g. estuaries and coastal zones) and temporal variations in contaminant concentration [12]. As such, bioindicators can give comprehensive information on the effects of the pollutant on the ecosystem compared to direct measurements of the physical/chemical parameters.

The assessment of ecological health in marine system is governed by two primary pieces of legislation, the Water Framework Directive (WFD) and the Marine Strategy Framework Directive (MSFD). The WFD is based on an ecological assessment rather than a traditional physico-chemical one, to assess the degree of degradation caused by anthropogenic pressures [13]. This degradation must be assessed using different biological quality elements (BQE). One of the BQE used to assess the Ecological Status (ES) in coastal and transitional water bodies is based on the composition and abundance of the marine macroalgae. The presence of ‘blooms of green opportunistic macroalgae’, mainly of the genus *Ulva*, have been primarily used for assessment of transitional waters in Ireland [14, 15] and other European countries [16]. The WFD assessment of opportunistic algae can also be considered when assessing the MSFD descriptor in Eutrophication (D5) in coastal waters. However, for these investigations the sites are transitional waters so outside to scope of the MSFD. The definitions of Good status differ between the two directives, with the Water Framework Directive (WFD) aiming for at least Good Ecological status and the Marine Strategy Framework Directive (MSFD) aiming for Good Environmental status. There are differences between the directives as to how such an environmental position is defined with the WFD focusing on Biological, chemical and supporting elements to describe status and the MSFD looking at a wider range of descriptors of the entire marine ecosystem. There are also differences in the scale of the assessment. The WFD is concerned with all surface and ground water bodies in member states up to 1 nautical mile from the land. The MSFD covers the entire marine waters of member states with the inner MSFD boundary incorporating coastal waters as defined under the WFD, but does not include the transitional waters (brackish and estuarine waters). In the coastal areas the overlap between the directives the MSFD is considered where it adds new elements not covered under the WFD.

Green macroalgae of the genus *Ulva* (commonly known as ‘Sea Lettuce’; Ulvophyceae, Chlorophyta) are cosmopolitan, able to tolerate a wide range of salinities and consequently are found in freshwater, estuarine systems and on open coasts [17]. Under favourable environmental conditions, such as elevated irradiance, raised water temperature, and reduced wave action [18, 19], *Ulva* spp. can thrive to such an extent that they become an environmental problem in the form of persistent blooms. Blooms may be stimulated or prolonged by anthropogenic nutrient inputs from agriculture, aquaculture, industrial and domestic waste [20, 21, 22]. Growth and accumulation of *Ulva* may result in anoxic decomposition and release of gaseous sulphur compounds (e.g. hydrogen sulphide, carbon disulphide, methyl sulphide). Exposure to these noxious gases can lead to health risks in both humans and wildlife [23]. Environmental impacts of the development and degradation of *Ulva* blooms include impacts on local biogeochemistry and biodiversity [24]. These negative influences on the local environment and the socio-economics of the affected region are often exacerbated by the scale of algal biomass being deposited. This can be illustrated by Brittany’s annual *Ulva* blooms where during 1992, 14,560 m^3^ of *Ulva* seaweed was removed from the shoreline at a cost of €1.8 million [24]. Similarly, the removal of over one million tonnes of green algal biomass in the Qingdao region of China during the 2008 *Ulva* blooms cost €200 million [24]. Although the economic impact can be significant, it has been suggested that managing the biomass could bring revenue to the local economy [25]. In this sense, this biomass could be used for animal feeds, fertilisers, and pharmaceuticals, among other uses [26]. However, the concentration of toxic metals could preclude these applications since it could pose a risk to human health.

Interspecific variations in growth and physiology can lead to different responses to environmental change, and differences in biomass quality for human uses (e.g. varying concentrations in ulvan, trace metals, and bioactive compounds). For instance, tubular species (which were formerly distinguished with the separate genus *Enteromorpha*) possess higher concentrations of trace elements than bladed taxa [27]. Furthermore, the presence of a combination of different species can also stimulate or prolong the intensity and duration of the bloom, since a temporal and spatial succession can occur [28]. The arrival of cryptic non-native species could explain the occurrence of macroalgal tides in places where nutrients conditions remain more or less constants [29]. For these reasons, the taxonomical clarification of the *Ulva* species that are forming green tides is key to understanding the occurrence of these blooms, and maybe to determine its possible applications. However, *Ulva* species are morphologically simple, but taxonomically complex: due to insufficiently reliable morphological characters to separate species [30, 31], a microscopic morphological identification often does not allow a definitive classification of *Ulva* species—molecular genetic information is needed to identify particular taxa, [30, 31].

The principal aims of the present study were: i) to assess the Ecological Status of different Irish transitional water bodies under different anthropogenic pressures based on the presence of ‘blooms of green opportunistic macroalgae’; ii) to establish the taxonomic identity of *Ulva* bloom-forming species; and iii) to ascertain the possible correlation between the status and metal content in the *Ulva* blooms. The study concludes with an evaluation of possible management strategies with the consideration of environmental and socio-economics implications (e.g. uses in animal feeds, pharmaceuticals, and fertilisers).

## Materials and Methods

### 2.1 Site selection

**Fig 1:**
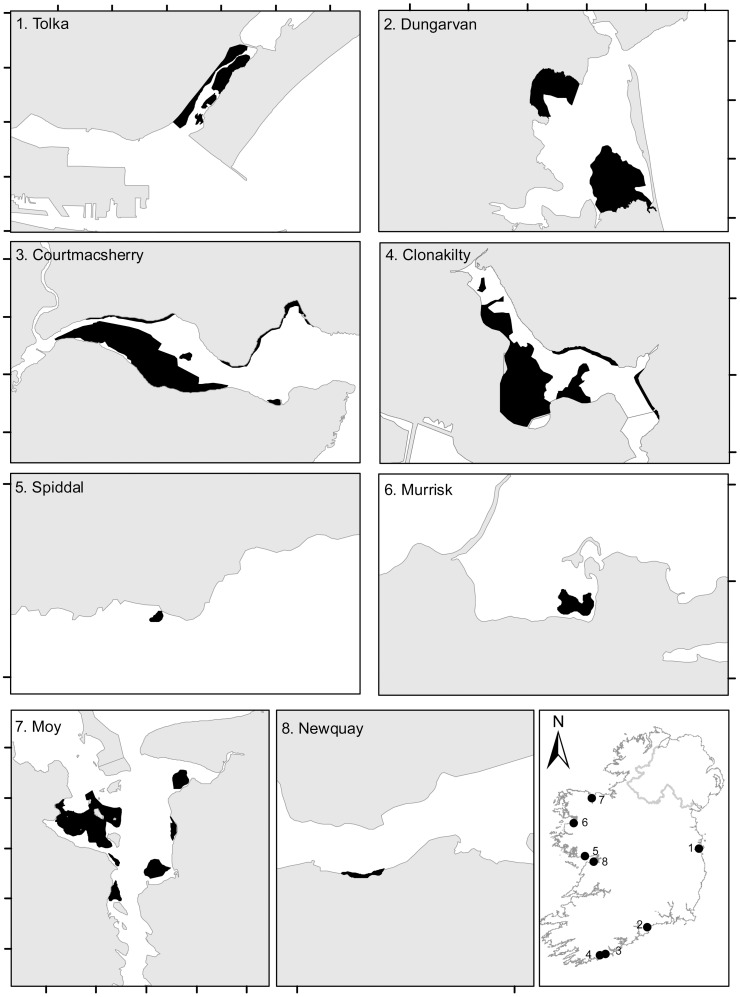
The location of Irish *Ulva* bloom sampling sites. Darkened areas on the map show the location of *Ulva* populations forming blooms. Tick marks on border are at 1km intervals.

The WFD requires EU member states to have a national monitoring programme for sampling and assessment of the biological quality elements (BQE) used for assigning ES [13]. As part of this programme, a series of water bodies are assessed for opportunistic macroalgal BQE on an annual or three-yearly basis [14]. A subset of eight Irish waterbodies representing both agricultural and urban pressures were selected for the present study (Fig 1). Water bodies were chosen in areas known to experience a dominance of bladed *Ulva* proliferations at differing scale of impact (i.e. excluding dominant tubular (enteromorphoid) bloom species, such as *Ulva compressa* L. and *U*. *intestinalis* L.) [32, 33, 34]. To minimise the effect of seasonal biomass variability, all samples were collected in the same month, August 2010. Specific permissions were not required for access or sampling and the species being investigated are not protected.

### 2.2 WFD assessment

The Irish WFD monitoring tool based on the abundance of opportunistic macroalgae is described by Scanlan et al. [16] and Ní Longphuirt et al. [15]. This tool considers spatial cover, biomass and persistence of algal growth to classify water bodies relative to undisturbed conditions [16]. Following WFD specification, the assessment provided by this tool is quantified into a single numerical value between 0 and 1, the Ecological Quality Ratio (EQR), which represents the ratio between the current and the reference (i.e. pristine or near-pristine) condition. Lower numbers lower ecological status (ES), so that EQR and ES are interchangeable. This consist of calculating the area of the intertidal available for growth of attached algae excluding areas such as channel edges, soft silt-banks and other areas not suitable for algal growth (Available Intertidal Habitat, AIH). This was undertaken using GIS prior to the field survey with desk-based assessments ground-truthed *in situ*. An initial assessment of the entire water body was undertaken to estimate roughly the areas of AIH affected by algal mats (the affected area, AA), and if algal cover (AA/AIH) exceeded 5% then a more detailed survey was undertaken. The outer edges of the patches of algae were mapped *in situ* using GPS. Multiple transects were taken through each patch depending on the overall size of the patch. A minimum or 1 and up to 4 transects were taken in each patch with a 500m separation between them. A minimum of 10 quadrats (0.25 m^2^) were placed haphazardly along each transect. The percentage cover and algal biomass in each quadrat was recorded. Cover was estimated by counting the number of squares in a 5x5 gridded quadrats filled with algae. Percentage cover was estimated in the field. Photographs of each quadrat were also taken for quality control checks in the lab after the survey. The presence of algae entrained into the sediment in each quadrat was recorded and total biomass was calculated by multiplying spatial cover by mean biomass. Assessments of cover and biomass were undertaken in situ at low water. The data were compiled into five sub-metrics to provide a WFD assessment for the water body and compared to the boundaries in Table 1.

1. Total percentage cover of the available intertidal area;
2. The lower of Total patch size of the affected area (AA), or the affected area (ha) as a percentage of the total available intertidal habitat (ha) (AA/AIH%);
3. Average biomass of algae on the available intertidal area;
4. Average biomass in affected area;
5. Percentage of quadrats with algae entrained into sediment.

**Table 1:** Boundary conditions for each of the algal bloom assessment sub metrics for calculated ecological status categories.

*Quality status*	%cover AIH	Biomass (g m^2^) AIH	Biomass (g m^2^) AA	%entrained AA	AA (ha)*	AA/AIH (%)*	EQR
Lower limit	0	0	0	0	0	0	**1.0**
High/Good	5	100	100	1	10	5	**0.8**
Good/Moderate	15	500	500	5	50	15	**0.6**
Moderate/Poor	25	1000	1000	20	100	50	**0.4**
Poor/Bad	75	3000	3000	50	250	75	**0.2**
Upper limit	100	6000	6000	100	6000	100	**0.0**

AA, affected area; AIH, available intertidal habitat.

*Only the lower of the two asterisked criteria is used in calculating the final overall water body Ecological Quality Ratio (EQR). EQR is synonymous with ecological status or ES. Adapted from Scanlan et al. [16].

Each sub-metric was scored with an EQR from 0–1 calculated as follows:

$$EQR=upper\;EQR\;range-\;\left\{\left(\frac{value-lower\;class\;range}{class\;width}\right)\times EQR\;band\;width\right\}$$

For example, if site χ % cover of 20 lies between 15–25, so

Upper EQR range = 0.6,

Lower class range = 15,

Class width = 25−15 = 10,

EQR band width = 0.2

$$EQR=0.6-\;\left\{\left(\frac{20-15}{10}\right)\times 0.2\right\}=\;0.5$$

The final Ecological Quality Ratio (EQR) was calculated as the average of all the sub-metrics.

The WFD tool is used to classify water into five Ecological Status categories (ESC: Bad, Poor, Moderate, Good and High) with boundary levels set on the basis of expert judgement and intercalibration exercises.

### 2.3 Sample collection

Materials for elemental analysis were collected from twenty 0.25m^2^ quadrats in each site, haphazardly placed across the algal patches. Three entire thalli without epiphytic attachments or signs of herbivory were collected within each quadrat. *Ulva* plants were individually placed into clean polythene bags, and stored in a cool box for transport to the laboratory. Samples were washed with Milli-Q water [18.3 MΩ·cm, Millipore, Bedford, USA] to remove debris and any adhering particulate material and then freeze-dried at -52°C [FreeZone 12, Labconco, Missouri, USA].

### 2.4 Molecular analyses

DNA was extracted from the freeze-dried material used for the metal analyses. Since this material consisted of mixed, small flakes, the extracted subsamples (10–20 mg) may potentially have belonged to more than one specimen. The extraction with a commercial kit [NucleoSpin^®^ Plant II, Macherey-Nagel, Düren, Germany] followed the manufacturer's instructions. The large subunit of the plastid-encoded Ribulose Bisphosphate Carboxylase-Oxigenase (RuBisCO) gene region (*rbc*L) was amplified in a polymerase chain reaction (PCR) using published primers SHF1 and SHR4 [31] at an annealing temperature of 55°C. In all other aspects, amplification, purification of PCR products and sequencing followed methods given in Heesch et al. [35].

To confirm the taxonomic identity of the "Certified Reference Material (CRM) for analytical quality assurance of '*Ulva lactuca'-* BCR-279" [European Joint Research Centre; Institute for Reference Materials and Measurements—IRMM, Geel, Belgium], a small scoop of the powdered material (~10 mg) was subjected to the same methods. Since the DNA of the CRM showed signs of degradation, its *rbc*L region was amplified and sequenced in two parts, using an internal primer pair developed for ancient *Ulva* samples (C. Maggs, personal communication). Care was taken not to contaminate the CRM by performing all procedures separately from field-sampled *Ulva*.

**Fig 2:**
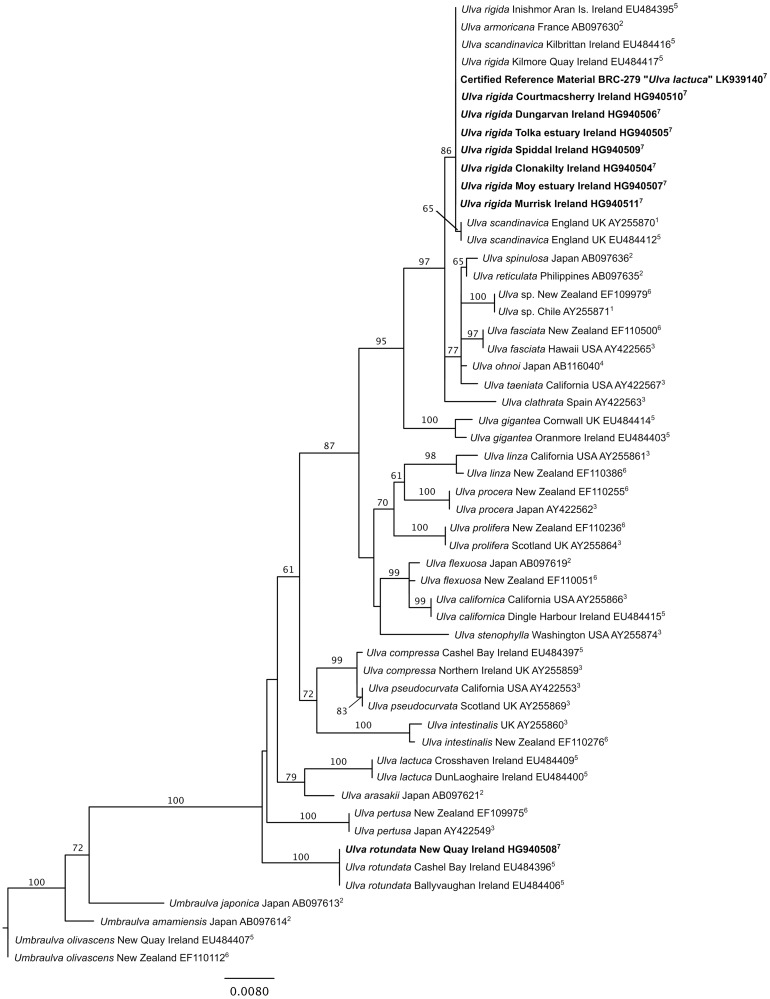
Phylogenetic tree inferred from a Maximum Likelihood analysis of partial *rbc*L sequences of *Ulva* species. Numbers above lines indicate bootstrap support values (branches without number received 50% support or less). Species names are followed by information on the sampling location, the GenBank/ENA accession number and the respective publication: ^1^: Hayden et al. [40]; ^2^: Shimada et al., [41]; ^3^: Hayden &Waaland [42]; ^4^: Hiraoka et al. [43]; ^5^: Loughnane et al. [39]; ^6^: Heesch et al. [31]; ^7^; present study, set in bold.

The resulting nine sequences were included in an alignment containing 46 published sequences, 42 representatives of the genus *Ulva* and four of the related genus *Umbraulva*, which served as outgroup (see Fig 2 for GenBank accession numbers and references). Sequence alignment and phylogenetic analysis under the Maximum Likelihood (ML) criterion followed Heesch et al. [35].

### 2.5 Sample acid digestion and metal measurement

Twenty pooled samples per sampling location (~200 mg of the three samples per quadrat) were digested in 1 mL 30% H_2_O_2_ [TraceSelect^®^ Ultra, Sigma-Aldrich, St. Louis, USA] and 5 mL 65% HNO_3_ [trace metal free grade, Fisher Scientific, Loughborough, UK] using microwave digestion [Multiwave 3000, Anton Paar, Graz, Austria]. The *Ulva lactuca* CRM [BCR-279] was used for quality assurance. There was a good match between the reported values for the CRM and the metal concentrations found in the current study (Table 2). Elemental (Al, As, Ba, Cd, Co, Cr, Cu, Mn, Mo, Ni, Se, Sr, Sn, Pb, Ti, V, and Zn) determination was performed using an Elan DRC-e Inductively Coupled Plasma—Mass Spectrometry, ICP-MS, [Perkin-Elmer, USA] [36].

**Table 2:** Observed results of the metal determination in certified reference material (CRM)-*Ulva lactuca* [BCR-279] found in the present study (mg kg^-1^, dry weight).

	Reported^1^	Found
*Certified values*		
As	3.09±*0*.*21*	3.24±*0*.*19*
Cd	0.27±*0*.*02*	0.24±*0*.*02*
Cu	13.10±*0*.*40*	12.38±*0*.*79*
Pb	13.50±*0*.*40*	13.17±*5*.*10*
Se	5.90±*0*.*40*	5.14±*1*.*79*
Zn	51.30±*1*.*20*	51.86±*9*.*49*
*Indicative values*		
Cr	10.73±*0*.*70*	9.61±*0*.*80*
Mn	2090.00±*50*.*00*	2245.07±*270*.*93*
Ni	15.90±*0*.*40*	13.90±*1*.*83*

^1^Reported values were derived from *Ulva lactuca* CRM no 279 BCR reference material report.

### 2.6 Statistical analysis

Statistical analyses were performed using PERMANOVA+ PRIMER 6 [Plymouth Routines in Multivariate Ecological Research], and SPSS software [V17, SPSS, IBM, Corporation, USA]. In all statistical analyses, significance was set at *p*-value <0.05, with randomizations based on 9999 permutations.

To assess differences in metal concentrations and composition between the different Ecological Status categories, a two-way Permutational Analysis of Variance (PERMANOVA) was carried out with ESC as a fixed factor, and Site as a random factor nested in ESC. In the case of metal composition, the dataset was initially normalised. All analyses were based on Euclidean Distance. Additionally, a distance-based test for homogeneity of multivariate dispersion (PERMDISP [37]), and a Principal Component Analysis (PCA) was performed to interpret and visualise the multivariate analysis of metal composition. Furthermore, Pearson’s correlations were used to quantify the associations between metals. To ascertain whether levels of metals found in seaweed were related to ecological quality ratios, Spearman’s correlation were used. Where correlation coefficients were averaged to summarize patterns, values were first z-transformed to reduce this bias in estimated means and confidence intervals [38]. Where PERMANOVA indicated significant differences among levels, these were explored (for a subset of potential toxic metals: V, Cr, Ni, Cu, As, Cd, Sn, and Pb) using univariate ANOVA.

## Results

### 3.1 Site assessment

Three sites were classified as high Ecological Status (Spiddal, Murrisk, and New Quay), three as moderate (Tolka, Dungarvan, and Moy) and two as poor (Clonakilty and Courthmacsherry). At the three localities of high status, the *Ulva* cover of the intertidal area was lower than 5%, and no biomass estimation was made at these sites. Full surveys were undertaken for the other five locations. The EQR score of these five locations ranged between 0.57 in Tolka and 0.38 in Courthmacsherry (Table 3). The survey revealed that the two sites categorised as ‘Poor’, with the lowest EQR scores in this study, i.e. Courtmacsherry and Clonakilty, had the largest algal blooms, with an estimated biomass of 2164 and 845 metric tonnes, respectively.

**Table 3:** Details of *Ulva* bloom sites and the designation of water quality in the study sites and estimated land use.

No	Site	Lat	Long	WFD	EQR	Spatial Cover (ha)	Mean Biomass (g m^2^)	Total Biomass (tonnes)
1	Tolka	-6.17233	53.36048	Moderate	0.57	40.63	1329.53	540.19
2	Dungarvan	-7.62002	52.07497	Moderate	0.54	108.71	302.50	328.85
3	Courtmacsherry	-8.72488	51.63683	Poor	0.38	128.78	1680.51	2164.16
4	Clonakilty	-8.86716	51.60880	Poor	0.38	76.12	1110.42	845.25
5	Spiddal	-9.31508	53.23941	High	1	<1	N/A	N/A
6	Murrisk*	-9.64144	53.78255	High	0.93	28.57	N/A	N/A
7	Moy	-9.15267	54.19604	Moderate	0.47	98.99	402.00	397.94
8	New Quay	-9.07403	53.15596	High	1	<1	N/A	N/A

WFD-Water Framework Directive status for algal biomass monitoring tool [16]. Five potential quality categories are used: ‘High’, ‘Good’, ‘Moderate’, ‘Poor’, and ‘Bad’. EQR- Ecological Quality Ratio- scale from 1–0, high is 1, and bad is 0 [15]. Spatial cover- Total area covered by algae, mapped in situ.

*Biomass was not assessed here as coverage was below WFD criteria for biomass assessment

### 3.2 Molecular genetic identification

The nine *rbc*L sequences produced in this study were between 1082 (sample from Tolka) and 1197 (*Ulva* CRM) bases long. Sequence comparisons showed that seven of the Irish *Ulva* blooms included in this study contained *U*. *rigida*, while the New Quay bloom comprised *U*. *rotundata* Bliding: the *rbc*L sequence from this site was identical to two other samples from sites in Galway Bay, Ireland identified as *U*. *rotundata* by Loughnane et al. [39], forming a 100% supported clade at the base of the *Ulva* ingroup in the phylogenetic tree (Fig 2). The *rbc*L sequences from all other blooms were identical or almost identical (a lower quality of the sequence from Tolka resulted in some unidentified bases, of which only two were in informative positions (thus equaling a potential 0.2% sequence divergence), while the others were in positions conservative to all *Ulva* species included in the alignment). These seven sequences comprised a well-supported (82%) clade at the top of the phylogenetic tree (Fig 2), together with GenBank sequences identified as *U*. *rigida*, *U*. *scandinavica* Bliding and *U*. *armoricana* P. Dion, B.de Reviers & G. Coat. The *U*. *rigida* clade also contained the sequence of the CRM, thus making the reference material a direct match for seven of the eight Irish *Ulva* bloom samples.

### 3.3 Metals concentrations

**Fig 3:**
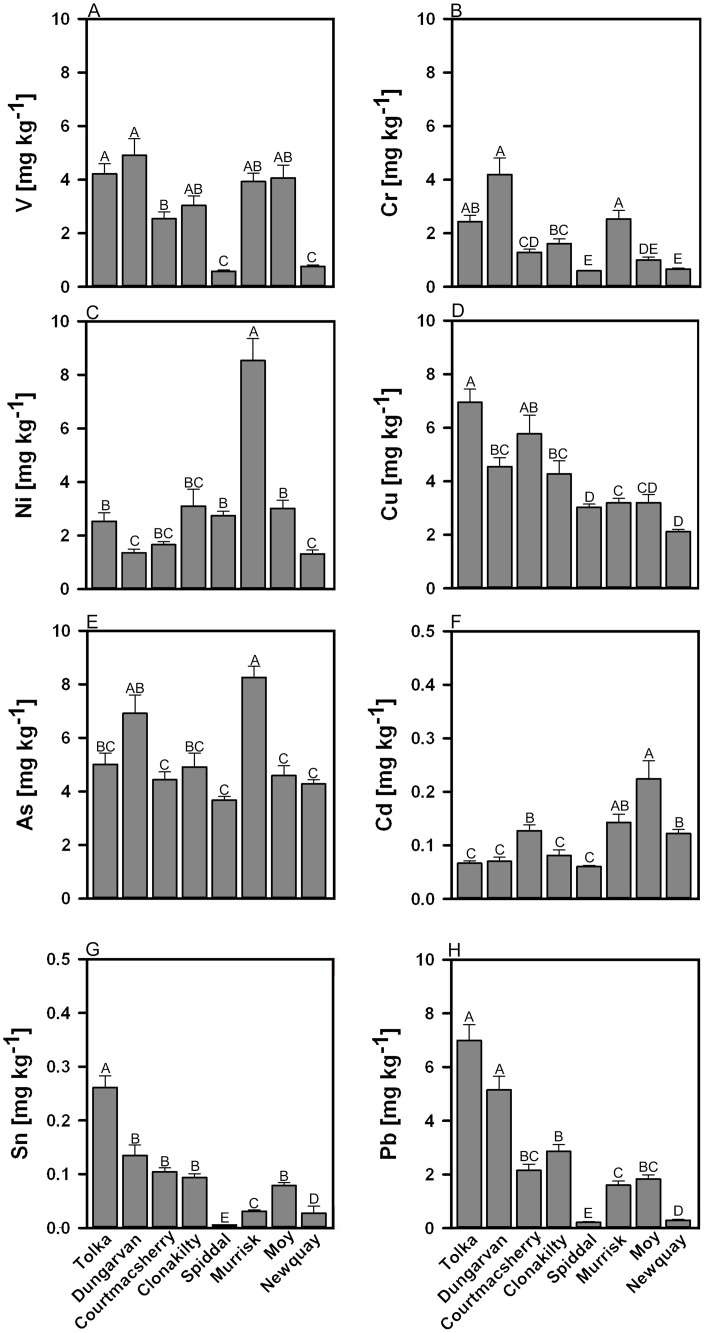
Mean concentrations of potentially toxic metals. (**A**) V, (**B**) Cr, (**C**) Ni, (**D**) Cu, (**E**) As, (**F**) Cd, (**G**) Sn and (**H**) Pb in *Ulva* blooms at the different sampling sites. ± S.E. Different superscript represents statistical significant differences (*P*<0.05).

*Ulva* metal concentrations are presented in Table 4 (Al, Ti, Mn, Co, Zn, Se, Sr, Mo & Ba) and potentially toxic metals in Fig 3 (V, Cr, Ni, Cu, As, Cd, Sn & Pb). PERMANOVA results for all metals showed that *Ulva* tissue concentrations differed significantly between different sites (*P*-values < 0.01, Table 5), but no significant effect in metal concentration was observed for ESC. *Ulva* from Dungarvan generally had the greatest metals concentrations in any site. While across all sites, Al occurred in highest quantities. *Ulva* from Dungarvan and Tolka contained significantly higher amounts of Al in comparison to other sites (*P*<0.001). The variability in Al contents between sites was also high, ranging from 23.45 ± *6*.*18* mg kg^-1^ in Spiddal to 1591.43 ±*294*.*72* mg kg^-1^ in Dungarvan.

**Table 4:** Metal content found in Irish *Ulva* blooms (mean ± SE, *n* = 20, mg kg^-1^, dry weight).

	Sites							
	Tolka	Dungarvan	Courtmacsherry	Clonakilty	Spiddal	Murrisk	Moy	New Quay
**Al**	1081.55±*116*.*80*	1591.43±*294*.*72*	548.75±*86*.*33*	509.79±*96*.*50*	23.45±*6*.*18*	336.81±*57*.*24*	234.93±*47*.*29*	90.58±*21*.*54*
**Ti**	23.52±*1*.*90*	25.29±*3*.*71*	13.11±*1*.*09*	18.38±*2*.*27*	7.62±*0*.*35*	13.09±*1*.*49*	14.48±*1*.*53*	9.08±*0*.*42*
**Mn**	128.56±*30*.*82*	131.56±*24*.*73*	212.06±*57*.*64*	144.57±*49*.*87*	25.58±*4*.*28*	125.95±*25*.*50*	20.71±*2*.*26*	15.81±*2*.*29*
**Co**	0.85±*0*.*05*	0.78±*0*.*07*	0.66±*0*.*06*	0.72±*0*.*09*	0.14±*0*.*01*	0.57±*0*.*04*	0.31±*0*.*02*	0.09±*0*.*01*
**Zn**	31.71±*1*.*71*	31.38±*3*.*11*	17.21±*1*.*18*	24.70±*1*.*61*	10.84±*0*.*31*	26.45±*2*.*40*	17.68±*1*.*37*	12.59±*0*.*34*
**Se**	2.67±*0*.*24*	2.94±*0*.*26*	2.92±*0*.*29*	3.05±*0*.*43*	5.53±*0*.*57*	4.04±*0*.*40*	3.93±*0*.*50*	2.51±*0*.*23*
**Sr**	71.19±*4*.*34*	97.33±*7*.*86*	91.72±*4*.*85*	114.70±*5*.*09*	90.93±*2*.*73*	94.45±*2*.*95*	78.00±*4*.*59*	73.99±*4*.*63*
**Mo**	0.22±*0*.*02*	0.13±*0*.*01*	0.10±*0*.*01*	0.14±*0*.*02*	0.11±*0*.*01*	0.10±*0*.*01*	0.07±*0*.*01*	0.09±*0*.*01*
**Ba**	5.83±*0*.*54*	5.98±*1*.*08*	3.22±*0*.*34*	6.51±*0*.*79*	0.30±*0*.*04*	4.24±*0*.*46*	3.72±*0*.*27*	1.59±*0*.*39*

**Table 5:** Permutational multivariate analysis of variance on the differences between ecological status and metal composition.

**PERMANOVA**	***df***	**MS**	**Ps-F**	**p (MC)**
Ecological Status	2	9.27 x10^6^	2.5433	0.1412
Site	5	3.65 x10^6^	10.777	*<0*.*0001*
Residual	144	3.38 x10^5^		
**PERMDis**	***p*-value**	**Ecological Status**	**Mean Dispersion**	
High-Moderate	*<0*.0001*	High	170.81±*19*.*90*	
High-Poor	*0*.0003*	Moderate	720.15±*90*.*83*	
Moderate-Poor	*0*.021*	Poor	392.65±*40*.*48*	

* indicates value has statistical significance (*P*<0.05), ± S.E.

*Ulva* collected from Courtmacsherry and Clonakilty had relatively low amounts of Cr, Pb, Sn, Se, and Zn, when compared to Tolka or Dungarvan blooms. By contrast, the levels of Cu, Cd, Mn and Sr were significantly (*P*<0.001) higher at Courtmacsherry and Clonakilty than at many of the sites, including Tolka and Dungarvan. The bloom in Moy had the highest recorded cadmium concentration (*P*<0.001, Fig 3F).

Spearman correlation coefficients between *Ulva* metal concentrations and site EQR were negative on average (Table 6, mean -0.43, CI -0.3272 to -0.5233). This implies a higher metal concentration at low EQR. However, there was variation between metals, with Se and Ag having correlations close to zero. Only Mn had a significant negative correlation with EQR (-0.735, *P* = 0.038). The evidence for generalizing patterns of metal concentration across EQR values is therefore weak and there was no evidence that potentially toxic metals were more or less strongly associated with EQR (F_1,19_ = 0.26, p > 0.05).

**Table 6:** Spearman’s correlation coefficient showing the relationship between metal concentration and ecological quality ratio (EQR).

Metal	EQR
Al	-0.554
Ti	-0.590
V	-0.337
Cr	-0.313
Mn	-0.735*
Ni	-0.193
Co	-0.530
Cu	-0.590
Zn	-0.277
As	-0.229
Se	0.084
Sr	-0.470
Mo	-0.193
Ag	-0.036
Cd	-0.327
Sn	-0.602
Sb	-0.627
Ba	-0.554
Tl	-0.313
Pb	-0.602
Bi	-0.602
**MCI**	-0.530

MCI, Metal Content Index is s calculated as the geometric mean of all metals concentrations,.

* indicates correlation value has statistical significance (*P*<0.05).

### 3.4 Metal composition

Metal concentrations relative to each other were not consistent across sites. Permutational dispersion analyses also showed differences in data dispersion between the ESC (*P*<0.05) (Table 5). The level of dispersion was greatest in the ‘Moderate’ category, while ‘High’ status had the lowest.

**Fig 4:**
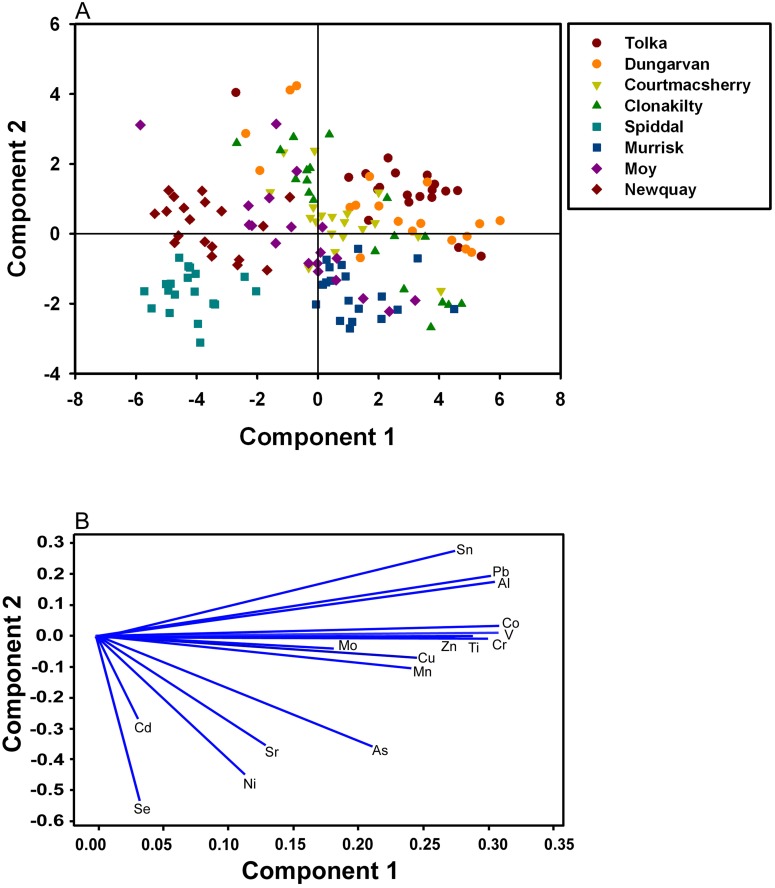
Principle component analysis. (**a**) Score biplot of the first and second principal component for metal concentration and sampled sites. (**b**) Loading plot of first and second principal component of the relationship between metals within all the sites. Component 1 and 2 explains 66.9% of the sample variation.

A principal component analysis (PCA) was conducted based on the metal levels from the eight sites (Fig 4). Over 66.9% of the variation between samples could be explained by the two principal components. The score plot showed that metal values found in Spiddal *Ulva* were closely clustered together (Fig 4A) due to lower metal concentrations. A similar clustering effect was observed at other sites including Murrisk, Courtmacsherry and Tolka; however, discrete groupings were less discernible due to overlapping score values with other sites. Weak clustering was found in the plotted scores for Clonakilty, Moy and Dungarvan, where high variations within each site’s score values led to a wider scattering over the score plot. The loading plot for all measured metals (Fig 4B) indicated that Se, Cd, Ni, Sr, and As did not correlate strongly with other metals (Pearson’s correlations, r<0.635). In contrast, metals such as, Al, Ti, Cr, V, Co, Sn, Ba and Pb generally correlated with one another (Pearson’s correlations, *p*<0.001, r>0.821). Furthermore, the plot highlighted two metals (Al, Sn) that were relatively closely associated with Pb (Pearson’s correlation, *p*<0.01, r = 0.871, and r = 0.901, respectively).

## Discussion

### 4.1 Algal bloom characteristics

One of the earliest reports of green algal blooms in Ireland was from Belfast Lough [44], and over the decades more occurrences were documented from other parts of the Irish coastline, including Dublin Bay [32], and Rogerstown Estuary [33]. More recently, blooms observed at Courtmacsherry and Clonakilty have been reported to have increased in severity [34]. In the present study, Courtmacsherry and Clonakilty had the largest algal blooms. In comparison to other blooms found in other parts of the world, the scale of the biomass produced in Ireland is dwarfed by both Brittany (France [24]) and Qingdao (China [25]).

These severe blooms may be linked to eutrophic conditions caused by nutrient loading from the surrounding catchments, with, for example, nitrate ranging between 9.170 and 26.50 mg L^-1^, respectively [45, 46, 47]. The high nitrogen loading has been implied by the highest stocking rate of dairy farms in Ireland [46] and this has been considered the main nutrient factor for affecting the *Ulva* bloom [15]. Similarly, nutrient enrichment linked to pig-farming was implicated in the *Ulva* blooms in Brittany [48], producing over 100,000 m^3^ of algae annually [24]. On the other hand, the presence of consistently high phosphorous concentrations (>0.056 mg L^-1^) throughout the year could be responsible for a substantial *Ulva* bloom in Tolka [45].

### 4.2 *Ulva* species composition

Previous studies have shown that *Ulva* blooms can be composed of one or several species [37, 49, 25, 28]. The presence of a greater number of bloom-forming species can favour the occurrence of blooms, prolong the duration, and intensity, since different species have different environmental requirements, leading to a temporal and spatial succession [28]. In this sense, the arrival of cryptic non-native species have been invoked to explain the occurrence of macroalgal tides in places where nutrients conditions remain constants or even improved, and macroalgal tides were not observed previously [29]. The molecular analyses suggest that the majority of the sampled blooms (seven out of eight) would be composed of *Ulva rigida* irrespective of the quantity of the biomass present. This suggested that the development of the blooms could not be attributed to interspecific differences in growth or ecophysiology between different *Ulva* species, but are more likely explained by environmental conditions, supporting the occurrence of *Ulva* as an indicator of water quality regardless of species affiliation. Nevertheless, the possibility that at some sites more than one species was present should not be discounted since only a specimen per locality was identified using molecular techniques.

The mislabelling of the *Ulva* CRM as "*Ulva lactuca*" by the provider [50], which was genetically identified in this study as *U*. *rigida*, highlights the importance of correct, preferably genetic, identification of any studied organisms including certified reference materials. In the present study, the misidentification of the CRM was inconsequential, as it comprised the same species as most of the studied blooms. However, errors from misidentifications of reference material could become problematic where studies confound species-specific variation with other factors.

### 4.3 Metal concentrations

The presence of elevated levels of macronutrients (e.g. nitrogen and phosphorus) in the water can often enhance metal accumulation in *Ulva*; laboratory experiments by Lee and Wang [51] showed enhanced uptake of cadmium in *Ulva fasciata* Delile when exposed to elevated nitrate, and greater accumulation of chromium with increased phosphate concentration. Enhanced elemental uptake is consistent with the nutrient-rich estuaries of Clonakilty and Courtmacsherry [46] where *Ulva* showed elevated metal concentrations in the present study. The lower levels of metals in *Ulva* at Spiddal may conversely reflect relatively low macronutrient input and trace element availability in the surrounding environment [46]. *Ulva* samples from the Tolka Estuary (Dublin), which typically receives industrial and domestic effluent from the surrounding urban environment [52, 53], had some of the highest levels of priority metal pollutants (Cu, Sn & Pb, Fig 4).

Relationships with anthropogenic input and metal content do not, however, explain all the patterns: *Ulva* samples from Dungarvan Bay had relatively high concentrations of potentially toxic trace elements (V, As, Cu, Cr & Pb), despite Dungarvan having less urban influences and lower estuarine nutrient levels [46]. A leather tannery operated at one time in this area, and the estuary had historically received effluents including metal salts such as chromium used in the tanning process [54]. It is possible that the detection of elevated metals in the algal bloom could be a response to these historic activities potentially due to metals accumulated in the sediment (Fig 3).

Murrisk also had significant amounts of cadmium compared to sites that receive more anthropogenic inputs. However, the high levels of cadmium measured at these sites may be geogenic in origin, with background release in the surrounding environment through weathering and erosion of bedrock [55]. This could also explain the significantly higher amounts of arsenic observed in *Ulva rigida* from Murrisk (Fig 3E) associated with the natural weathering of arsenopyrite in the quartz veins in the surrounding mountainous region [56, 57]. This can lead to elevated arsenic concentrations in the water that flows into the nearby embayment (Clew Bay [58]). This was similarly demonstrated by arsenic levels in *Ulva rigida* collected from the Gulf of Thessaloniki, NE Greece, where measured values correlated with those found in the sediment samples [59] highlighting the potential influence of local geogenic sources on concentrations of metal in *Ulva*.

Levels of metals in *Ulva* measured in the present study were comparable to those found in *Ulva* blooms associated with the Moroccan phosphate mining region [60]. On the other hand, the metal concentrations (Al, Cr, Mn, Co, Ni, Cu, Zn, Cd & Pb) in the Irish *Ulva* tended to be at the lower limits of those reported in *Ulva* from the Venice Lagoon [61]. In contrast, *U*. *rigida* collected near industrial and sewage outflows (Turkey [62]) had lower amounts of copper, zinc and cadmium, when compared to the current study.

### 4.4 Relationship between ecological status assessment and metals

The work conducted in the present study revealed that there was a generalised negative trend (20 out of 21 metals) between ES and metal composition; however, most of these correlations were not significant. The indices developed in the context of the WFD have focused on the assessment of eutrophication effects at the community level, since this pressure was identified as the most important threat for European aquatic ecosystems [16]. Although high nutrient and metal concentrations are frequently correlated due to their anthropogenic origin [63], there is not a causative relationship between these pollutants. The origin of anthropogenic sources of contamination (agricultural or industrial) in a specific area strongly influences the nutrient/metal ratio and the elemental composition of inputs. While some activities such as agriculture will produce effluent with a high nutrient/metal ratio, industrial activities are expected to produce effluents with a lower nutrient/metal ratio with a very specific metal signature depending on the activity [64]. Moreover, the variability due to anthropogenic activities must be considered alongside the variability associated with natural geogenic sources, e.g. in the metal composition in *Ulva* in Murrisk. While the PERMANOVA results based on metal concentrations and composition failed to reveal a relationship with ESC, the PERMDISP analysis and the PCA indicated that sites with moderate and poor ESC had a higher dispersion in metallic composition in comparison to sites classified as high ESC. This suggested that data dispersion for tissue metal content of *Ulva* may be an indication of this anthropogenic pressure. Nonetheless, a relationship between ES and metal contamination could not be fully established in the present study. However, the determination of *Ulva* metal concentrations provided additional useful information on the status of marine communities, which is important for a wider understanding of the anthropogenic pressures in the coastal environment. Furthermore, this information is essential, if the *Ulva* biomass is considered for commercial exploitation.

### 4.5 The environmental and socio-economics of *Ulva* blooms

In order to limit environmental and economic degradation to the affected areas, a number of approaches have been suggested to obviate the problems associated with large mats of decomposing *Ulva* along the shore. These strategies involve either removing biomass to landfill or finding an alternative use for the biomass, such as animal feedstock, bioplastics, or biofuel production [65, 66, 67]. Removal of biomass to landfill or other disposal can incur large costs from transport and labour to mechanical collection [68]. Calculations by the Irish government-established Sea Lettuce Task-Force [34] estimated the cost of disposal of *Ulva* blooms on arable land at €16 per tonne compared to a landfill disposal cost of €260 per tonne. Using the estimated cost of landfill disposal, the expenditure required for removing the most severe blooms from this study would cost the following: Courtmacsherry €562,682, Clonakilty €219,765, and Tolka €139,516. On the other hand, disposal on arable land would amount to a total value of €56,737 (€34,627, €13,524, and €8,586) for the three affected sites.

Landfill and composting have been used as a means of disposal for *Ulva* blooms in France [24] and China [69]. However, the present study emphasises that bloom biomass may contain relatively high concentrations of potentially hazardous metals suggesting that such disposal methods may have other implications. Decaying *Ulva* could leach metals over time into a landfill site or over agricultural ground, and thus contaminate the surrounding soil, groundwater, or surface waters. This was evident from the risk of groundwater arsenic contamination following the application of seaweed (*Ascophyllum nodosum* (L.) Le Jol) fertiliser in a golf course setting [70]. The estimated total algal biomass from Tolka (540.19 tonnes, ww; 160 tonnes, dw, (if ww moisture level is 79.6% [71]; Table 3) could potentially comprise the following metals if the entire bloom was collected: V- 0.46 kg, Cr- 0.37 kg, Ni- 0.38 kg, Cu- 1.05 kg, As- 0.76 kg, Cd- 0.01 kg, Sn-0.04kg and Pb-1.06 kg (calculated from *Ulva* dw biomass x metal concentration). The algal bloom in Courtmacsherry (2164.16 tonnes, ww; 441.49 tonnes, dw) could contribute: V- 1.06 kg, Cr- 0.54 kg, Ni- 0.70 kg, Cu- 2.43 kg, As- 1.86 kg, Cd- 0.05 kg, Sn-0.04 kg and Pb- 0.90 kg. While composting *Ulva* on agricultural land may be more cost-effective than landfill [34], the increase in soil metal content from *Ulva* disposal could lead to the potential contamination of crops increasing the probability of human exposure [72]. There are many factors that could influence the quantities and impact of metals released into the surrounding soil, including: land surface area used for algal disposal, interaction between the leached metals and surface soil and precipitation levels. The number of variables precludes any robust modelling of impact and highlights the possible implications of disposing the algal biomass on land, particularly when the same land is repeatedly used for algal disposal, if the collected biomass increases, or metal accumulation in the *Ulva* bloom was enhanced. Further research is required to elucidate the effects of seaweed disposal on surface soils and to provide better risk assessments for disposal of *Ulva* blooms, particularly in relation to NaCl and other salts, and their effects on soil structure [34].

Many EU and other international regulations that recommended maximum allowable limits for metals in foods, feeds and seaweeds as a food source refer only to total metal concentrations [34, 73, 74] (Table 7). In general, the blooms sampled in this study complied with most EU and Canadian regulations for metal concentrations in animal feeds (2002/32/EC [73] and RG-8 regulatory guidance [75]; contaminates in feed). Data from the Moy showed that the mean cadmium levels exceeded a Canadian regulatory limit of 0.2 mg kg^-1^ for equine feeds, but not for other livestock (0.4 mg kg^-1^ [76], Fig 3 and Table 7). A comparison between the metal levels found in the algal blooms and the legislative limits for seaweeds in Australia, New Zealand and the European Union revealed As, Sn, Cd, and Pb were within acceptable regulatory tolerances [77, 78, 79]. In contrast to animal feeds, legal metal thresholds in foods for human consumption are lower, and consequently many of the metals determined in Irish *Ulva* blooms exceeded these limits (albeit with some extrapolation for cases where no specific ‘seaweed’ limit is defined). One example is Pb, where concentrations were higher than the international legal limits (0.1–2 mg kg^-1^) for seafood, terrestrial animals, and plant materials [77, 76, 79, 80, 81]. Scientific knowledge on safe consumption limits and toxicological effects of metals in seaweeds remains relatively unexplored, and many of the safe tolerances of metals (other than Cd, As, Sn and Pb) are still undefined by legislation and remained unregulated. The present study showed that many of the surveyed sites could potentially be used for direct and indirect human consumption.

**Table 7:** International legislative/recommended maximum limits of toxic elements in foods and animal feeds (mg kg^-1^, dry weight).

	Cd	As	Sn	Pb	Reference
Australia and New Zealand					
Fish		2*	250	0.5	[79]
Crustacean and Molluscs	2	1–2	250	2	
Seaweed		1*	250		
Vegetables and Plants	0.1–0.5		250	0.1–0.3	
Canada					
Animal Feeds	0.2–0.4	8		8	[76,82]
Fish protein		3.5		0.5	
Plant & Vegetable		0.1		0.2–1.5	
European Union					
Food (general)			0.20		[75,77, 78]
Bivalve	1–3			1.5	
Crustacean	0.5			0.5	
Fish	0.05–0.3			0.3	
Plants & Vegetables	0.05–0.2		0.02	0.1–0.3	
Seaweed	3.0	40(0.2*)		10	
Calcareous algae		10			
Animal Feeds and feedstuff	1–10	4–10		5–40	
FAO					
Fish and meats			50–250	0.1	[81]
Plants and Vegetables	0.05–0.4	0.1	250	0.1–1.5	
Crustacean and Mollusc	2				
Hong Kong & China					
Fish and shellfish	2	6–10	230	6	[80, 83]
Plants and Vegetables	0.1	1.4	230	6	
General foods (wet weight)	0.2			0.3	

* refers to inorganic arsenic.

## Conclusion and Recommendations

The present assessment and characterisation of Irish green tides has shown that the majority of bladed *Ulva* populations forming the blooms were *U*. *rigida*. The greatest bloom biomass was recorded at Courtmacsherry and Clonakilty. While the highest metal concentrations were generally associated with lower bloom EQR values, no clear link between the algal WFD assessment criteria and metal content was established. In general, based on international regulations concerning algal tissue metal content, the blooms surveyed were still suitable for commercial exploitation. Further development of uses for algal bloom biomass depends on the specific application and the site-specific profile of metal concentrations. *Ulva* blooms are becoming a regular and increasingly severe problem in estuarine systems and coastal seas, and the present assessment and characterisation of algal blooms will inform decision-makers and policy regarding in the management of green macroalgal blooms.
